# Starting conversations about mental health and wellbeing in Australian culturally and linguistically diverse communities

**DOI:** 10.1093/heapro/daae099

**Published:** 2024-08-13

**Authors:** Alyssa R Morse, Dianna G Smith, Rosemary Clifford, Brad Shrimpton, Michelle Banfield

**Affiliations:** Centre for Mental Health Research, National Centre for Epidemiology and Population Health, ANU College of Health and Medicine, The Australian National University, Canberra, Australian Capital Territory, 2600, Australia; Centre for Mental Health Research, National Centre for Epidemiology and Population Health, ANU College of Health and Medicine, The Australian National University, Canberra, Australian Capital Territory, 2600, Australia; Mental Illness Education ACT, Genge Street, Canberra City, Australian Capital Territory, 2601, Australia; Mental Illness Education ACT, Genge Street, Canberra City, Australian Capital Territory, 2601, Australia; Centre for Mental Health Research, National Centre for Epidemiology and Population Health, ANU College of Health and Medicine, The Australian National University, Canberra, Australian Capital Territory, 2600, Australia; The ALIVE National Centre for Mental Health Research Translation, The University of Melbourne, Grattan Street, Parkville, Victoria, 3010, Australia

**Keywords:** multicultural, culturally and linguistically diverse, CALD, mental health and wellbeing, co-production, lived experience

## Abstract

Australia is a multicultural nation with nearly 30% of the population born overseas. Migrants’ mental health can be impacted by discrimination, racism and experiences relating to asylum and immigration. These can be compounded by low help-seeking caused by stigmatized beliefs and poor mental health literacy. My Mind, My Voice (MMMV) is a co-designed program aiming to promote awareness of mental health and wellbeing for people with a culturally and linguistically diverse (CALD) background. This research project explored the perceived impacts and value of MMMV and processes leading to those impacts. A mixture of internal quantitative and qualitative evaluation surveys (*n* = 32) and researcher-conducted semi-structured interviews (*n* = 9) were conducted with CALD organization and community members who attended training workshops, presented MMMV events or attended an event. Data were analysed using a reflexive thematic analysis approach. Five themes were developed: *culturally relevant and respectful*, *cross-cultural connections*, *the importance of language*, *increasing confidence and literacy* and *the potential to change attitudes*. Being involved with a co-produced program that was culturally relevant and respectful was a positive experience that enhanced people’s confidence and literacy. Feeling respected, valued and validated helped participants feel empowered to develop and deliver mental health and wellbeing education in their community. Open, honest conversations are an important way to break down stigma and start conversations about mental health and wellbeing in CALD communities. Evaluation outcomes demonstrate the success of MMMV’s collaborative approach, which can inform the development and evaluation of CALD mental health promotion interventions.

**Contributions to Health Promotion**

- A co-produced program provided opportunities to learn skills and knowledge that could enhance mental health and wellbeing education in culturally and linguistically diverse communities.
- Participants reported improved confidence and health literacy after being involved in a program that provided a safe, culturally appropriate space to learn.
- Participants felt that they were empowered to develop and deliver mental health and wellbeing education in their communities after participating in this program.
- Channels of communication both within and between communities were opened for participants in this program.

## BACKGROUND

In 2021, Mental Illness Education ACT (MIEACT), the Australian Capital Territory’s (ACT) primary mental health and wellbeing education and training provider, initiated a project titled *My Mind, My Voice* (MMMV). This project was co-designed to increase awareness of mental health and wellbeing for people with a culturally and linguistically diverse (CALD) background. MIEACT provided opportunities for community members to learn the skills and knowledge to assist their community to find culturally appropriate and inclusive ways to enhance mental health and wellbeing (Mental Illness Education ACT, 2023a). This article reports on the analysis of qualitative data collected in an evaluation of the program.

Australia is a multicultural nation with nearly 30% of the population born overseas, equating to around 7.5 million people (Australian Bureau of Statistics, 2022a). All migration decreased during 2020–21, with the largest group of migrants arriving from Oceania during this time. Before the pandemic, most immigrants came from South and Central Asia (28% of migrants coming to Australia), compared to a decade ago 2010–11 when the largest migrant groups came from Northwest Europe (20%) (Australian Bureau of Statistics, 2021). Government estimates of permanent migration between 2000 and 2016 show that over half of the people coming to Australia were here as part of the skilled migration program, with the largest percentage of that group coming from India. The largest group of family migrants came from mainland China, and people from Iraq or Afghanistan were the largest group of migrants arriving with a humanitarian visa (Australian Bureau of Statistics, 2018). In 2016, people in Australia identified over 300 different ancestries and spoke over 300 different languages (Australian Bureau of Statistics, 2017). Collectively, people from such diverse backgrounds are grouped together under the term ‘culturally and linguistically diverse’ (CALD) in the Australian context.

The World Health Organization defines mental health as ‘a state of well-being in which the individual realizes his or her own abilities, can cope with the normal stresses of life, can work productively and fruitfully and is able to make a contribution to his or her community’ (Herrman *et al*., 2005). They maintain that mental health is an integral part of health, is more than just an absence of mental illness, and that mental health can be protected or weakened by individual, family, community and structural factors (World Health Organization, 2022). Among CALD communities, experiences of racism, stress from migration, asylum and settlement and poor mental health literacy can contribute to higher risks of mental illness. Blignault *et al.* (2022) outline further factors that can affect migrants’ mental health including, difficulties securing meaningful employment (particularly when education or qualifications are not recognized), changes to traditional gender roles, intergenerational family disagreements and discrimination or stigma. At the same time, official statistics habitually show that fewer people born overseas report mental health issues, compared to those born in Australia (Australian Bureau of Statistics, 2022b). Due to inadequate epidemiological data, it is difficult to know if CALD people underutilize mental health services and how many are assisted with their mental health (Minas *et al.*, 2013). However, research has shown that there are low rates of service use and higher rates of involuntary admissions in ethnic communities (Blignault *et al.*, 2009).

Some of the barriers or difficulties that can arise to stop or limit the use of mental health services are: different concepts of mental health (Colucci *et al.*, 2015; Wohler and Dantas, 2017); stigma; health literacy; lack of bilingual practitioners and resources (Blignault *et al.*, 2009) and language barriers including women being uncomfortable discussing personal issues in a foreign language, and not being able to articulate properly in a second language (Wohler and Dantas, 2017). Other reasons for the underutilization of health services are the perception of the appropriateness of seeking help, trust and negative past experiences (Colucci *et al.*, 2015), perceived safety of community resources (Vera and Conner 2007) and perceived discrimination (Telenta *et al.*, 2020).

Blignault *et al.* (2009) concluded that a variety of strategies were needed to increase the utilization of services and suggested that the co-production of these services was essential. Several studies have shown that there is also a great need for increased availability of culturally appropriate professionals and resources (Chan, 2009; Colucci *et al.*, 2015). According to Khawaja and colleagues (2013), it is important to ensure that health services and professionals advertise their services and this information is sent out to the appropriate people, including community leaders, elders and trusted community services.

Social capital in the form of family and friends, values and spirituality is also vitally important in managing mental health and illness (Moos, 2003; Chan, 2009). Using a trusted elder to normalize mental health and initiating opportunities where people could share their stories in a group session made people feel less isolated; they were more likely to feel that it is ‘not just me’ (Vera and Conner, 2007; Colucci *et al.*, 2015). Implementing these strategies in a culturally appropriate and supportive way would require appropriate and targeted education for elders and role models (Colucci *et al*., 2015). Effective community engagement in identifying and testing the right messaging is needed, not just to increase participation in services and activities but also in trying to influence wider attitudinal change (Telenta *et al.*, 2020).

MIEACT have run evidence-informed educational programs utilizing volunteer educators living with mental health issues for 30 years. MIEACT’s programs are facilitated by volunteer educators who use their lived experience of mental ill-health (personally or as a carer) combined with evidence-informed content to raise mental health understandings, address stigma and allow others to see themselves in their stories (Mental Illness Education ACT, 2022). These programs are designed to bring small audiences into direct positive contact with people living with mental illness, providing a platform to share experiences and educate the audience on what it is like to live with a mental illness to promote help-seeking (Mental Illness Education ACT, 2022). MIEACT’s programs are underpinned by the DoNOHarm framework which is a set of six principles that seek to ensure safe story sharing by avoiding language and imagery that can be triggering and traumatizing to lived experience story sharers and audiences (Mental Illness Education ACT, 2023b). Educators are trained in the MIEACT’s DoNOHarm Framework principles, story crafting and facilitation and learn from other educators by observing their presentations in the programs. Ongoing support is provided to the volunteer educators by MIEACT staff.

This study explored the perceived impacts and value of MIEACT’s MMMV program and the processes that led to those impacts. Data collection and analysis placed particular focus on exploring the cultural relevance, safety and inclusivity of MMMV and the practices that facilitated these characteristics. This article reports on the analysis of qualitative data collected in our evaluation of the program.

## METHODS

### My Mind My Voice program

The MMMV program aims to promote mental health and wellbeing in CALD communities in the ACT. MMMV has provided training and support to local partner organizations and community groups to facilitate engagement in a wide range of mental health-promoting activities. To assist partner organizations, community members were supported in attending two MIEACT training workshops, DoNOHarm and Story Crafting, to prepare them to present mental health and wellbeing content. These workshops build skills for talking about experiences of mental illness using inclusive language, minimizing referred trauma and setting personal and professional boundaries (Mental Illness Education ACT, 2023b). A range of mental health promotion events and resources for CALD communities were also co-produced by partner organizations, community members and MIEACT. Examples of these included mental health and wellbeing videos (Mental Illness Education ACT, 2023c), help-seeking flyers and lived experience-informed information sheets (Mental Illness Education ACT, 2023a), expos, market stalls, community presentations and My Mind My Voice: Stories of People from Around the World podcasts (Mental Illness Education ACT, 2023d).

### Evaluation design

To minimize the impact of the evaluation on community engagement with the MMMV program, evaluation tools were developed in consultation with MIEACT staff and were designed to present minimal participant burden in terms of time and effort. Partner organization perspectives on the evaluation design were gathered by MIEACT staff and relayed to the research team. This information included preferences for format (e.g. survey vs interview) and language (e.g. use of the phrase ‘mental health’ vs ‘wellbeing’). The independent evaluation used a mixture of internal evaluation survey data collected via collaboratively developed evaluation tools and researcher-conducted semi-structured interviews with program participants to explore the value and perceived impact of the MMMV program and identify key underlying processes.

### Ethical approval

The ethical aspects of the research were approved by The Australian National University Science and Medical Delegated Ethics Review Committee (protocol numbers 2021/132 and 2020/443). All interview participants gave written informed consent before interviews were conducted. Ethical approval was granted to perform a secondary data analysis on survey data collected by MIEACT; participants were informed that survey data were collected for the purpose of evaluation.

### Participants

Three key groups of participants were identified for this study, representing the variety of ways people engaged with MMMV:

1. multicultural organization and community members who attend the DoNOHarm and Story Crafting workshops (training workshop participants, Table 1),
2. people who presented or organized MMMV sessions and events (event organizers, Table 1), and
3. community members who attended an MMMV session or event (community event audience members, Table 1).

**Table 1:** Evaluation participants (*N* = 41)

Participant group	Data collection method	*n*
*Interview participants*		*N* = 9
MMMV program participants	Semi-structured interview	7
MIEACT interns	Semi-structured interview	2
*Survey participants*		*N* = 32
Community event audience members	Evaluation survey	1
Training workshop participants		26
	Evaluation survey: DoNOHarm and Story Crafting	15
	Feedback survey: DoNOHarm	8
	Feedback survey: Story Crafting	3
Event organizers	Evaluation survey	5

**Note**: Evaluation surveys were designed collaboratively by MIEACT and the research team and administered by MIEACT as part of the MMMV evaluation. Feedback surveys were designed and administered by MIEACT before the evaluation formally commenced.

Nine semi-structured interviews were conducted. Participants included seven MMMV program participants and two MIEACT interns from diverse communities who worked on the MMMV project. Interview participants had experienced multiple elements of the MMMV program, including attending a DoNOHarm and/or Story Crafting workshop, developing and/or delivering a community event or resource or participating in an event. The semi-structured design of the interview protocol allowed all relevant experiences to be explored with participants as needed. A total of 32 surveys with qualitative data were collected by MIEACT and provided to the research team (Table 1). This included data from the evaluation surveys, and additional feedback survey data collected prior to the evaluation commencing.

### Recruitment and informed consent

MIEACT staff members distributed recruitment emails to people who had participated in an MMMV training workshop or event to provide a warm introduction to the study. Potential interview participants were asked to contact the ANU research team directly, and informed consent processes were conducted by the ANU research team. Survey participants received a link to complete evaluation or feedback surveys directly from MIEACT staff. Completion of the surveys was considered consent for data to be used in research activities.

### Survey procedure

Following consultation with MIEACT and their partner organizations, three brief, bespoke post-session surveys were developed to gather descriptive information on the experiences and impacts of MMMV for each key participant group. Using a mix of qualitative and quantitative questions, the surveys explored participants’ overall impressions of the workshop or event they attended or organized, its cultural relevance, representativeness and inclusivity, and its perceived impacts on knowledge, confidence to present mental health content or seek help and community attitudes. Evaluation surveys were administered online by the MIEACT team using the Survey Monkey platform.

### Interview procedure

All participants were given the choice of having the interview conducted face-to-face, or remotely via videoconferencing software or over the phone. One interview was conducted face-to-face, the remaining eight were conducted remotely (1 via phone, 7 via videoconference). Interview questions were adapted to suit participants’ involvement in MMMV and explored survey topics in-depth with an additional focus on whether participants felt heard and respected during program activities, and current wellbeing concerns in CALD communities. Interviews were conducted by ARM or DGS, audio-recorded and professionally transcribed verbatim.

### Data analysis

Data were managed using QSR International’s NVivo 12 Pro software. All interview transcripts and survey responses were imported into the software. Data analysis followed a reflexive thematic analysis approach (Braun and Clarke, 2006). Interviews and open-ended survey responses were deductively and inductively coded. Initially, qualitative responses were deductively coded based on the objectives set out in the funding agreement for the MMMV program (Table 2). Objectives were intended to be assessed via participant self-report. A second stage of inductive coding was performed to gain broader insight into the perceived impacts and value of the program. Through this process, codes connected by shared meanings were developed into higher-order themes. The initial coding was performed by one author (D.G.S.) and was then refined into higher-order categories by a second author (A.R.M.). The research team regularly discussed the analysis process to reflect on decisions and clarify meaning. Discussions included input from a MIEACT staff member (R.C.) who identified key issues that arose during program development and implementation, these issues were followed up in subsequent interviews. Interviewers also kept reflective notes and pursued arising relevant topics with future participants. For example, an observed community partner’s need for more time to feel comfortable using new skills and knowledge was highlighted in these discussions and followed up in subsequent interviews.

**Table 2:** Deductive coding categories based on program funding objectives (objective target in brackets)

1	Culturally sensitive, responsive and significant for the wider multicultural community within the ACT (program).
2	Representative of participants’ cultural community groups (program).
3	Increased confidence to deliver peer-led mental health education within representative communities (participants).
4	Increased mental health literacy (participants).
5	Increase in mentally health behaviours (participants).
6	Increase in positive attitudes within community groups regarding experiences of mental ill-health (participants).

## RESULTS

Five themes were developed through the process of deductive and inductive coding, describing the impact and value of MMMV and the processes that led to that impact. *MMMV is culturally relevant and respectful*, providing a safe and collaborative space for learning, sharing ideas and developing skills. Participants’ experiences highlighted *the value of cultural and community connections*, and *the importance of safe and accessible language* when developing and delivering mental health and wellbeing education in CALD communities. Participants who engaged with MMMV experienced *increased confidence and mental health literacy* and observed current and potential future *positive impacts on personal and community attitudes*. Each of these themes is discussed in detail below.

### MMMV is culturally relevant and respectful

Participants expressed strong agreement that MMMV was a program that respected the views of participants and their community. The training workshops provided a culturally appropriate and safe space for learning about mental health and wellbeing. Facilitators modelled safe storytelling and created an environment where participants felt able and encouraged to discuss their own experiences and concerns.

…*the facilitator was speaking from their own experience as well and sharing their story, and just role modelled that sense of this is a safe space, I feel safe to share about myself, I hope you feel safe too....when the scene is set at that level, and that level of vulnerability then others were able to open up. I think that did make a difference*. (Interview 3)

*The workshops focused on the experiences of community members and allowed participants to share their personal thoughts and opinions without judgement.* (Training Workshops Survey Participant 8)

Participants also valued the recognition and respect for different views and opinions demonstrated by the MMMV program, training workshop facilitators and other participants. Many participants described their interactions with facilitators and other workshop attendees as respectful, non-judgemental and an opportunity to learn from each other.

…*I feel respected, I feel listened to, I feel that they were there also to hear us rather than just deliver....I think that set the tone and the expectations around the workshop, because we were no longer expecting to receive information, the whole table was there to exchange ideas and receive information.* (Interview 6)

*Everyone was given the opportunity to speak when they wanted to and no judgements were passed. It was also a really great way to connect with other members of my community.* (Training Workshop Survey Participant 1)

Participants appreciated that MMMV allowed them the space to learn about mental health and wellbeing without being pressured into doing things a specific way. They felt that MMMV provided an opportunity to build knowledge, skills and resources for community-led mental health promotion without community needs being overshadowed by the objectives of an external organization. MMMV was described as providing support without pressure and a safe space to learn about mental health and wellbeing. Participants reported that they had left the workshops with skills and tools to talk about sensitive topics (e.g. suicide) safely.

Workshop facilitation and the storytelling-based approach to communication were described as relevant to and appropriate for participants’ own communities and the broader multicultural community. Storytelling was seen as a powerful form of communication that created a sense of connection, empathy and trust, and encouraged engagement much more effectively than presenting facts and figures alone.

*…my culture specifically has a lot of focus on community, on communication, on storytelling and that’s what best for us, and that’s what works best in sharing knowledge between people. Which is why MIEACT’s focus on storytelling is a really important thing, and a really valuable thing for us to do. But also to have that space to adapt it for everyone who needs it.* (Interview 2)

Many interview participants highlighted the need for mental health and wellbeing resources that are culturally appropriate and accessible. Some participants noted that westernized resources could discourage engagement ‘*because you looked at a flyer and it didn’t look like you*’ (Interview 1). Participants described the value of increasing the representativeness of available mental health resources, allowing people to see themselves in the stories and experiences of others. It was also important to communicate in the most suitable way for the intended community and disseminate information through outlets that are easy to find and access. Information that was tailored for one community, may need to be adapted or re-designed to work for another.

*It is that really important thing of finding the ways to communicate that actually work for the people you’re talking to, rather than that, we either have things too complicated or hard to find and access, the amount of information that’s out there in weird inaccessible places.* (Interview 8)

### The value of cultural and community connections

Some participants reflected on the lessons that could be learned between communities, or between different generations within a single community, through the MMMV program. While each multicultural community in the ACT had its own specific needs and views on mental health, these participants noted that there were similarities in the experiences of people from different backgrounds. One lived experience story could resonate with people from multiple communities, increasing the potential impact of each MMMV presenter, event or resource. Sharing stories across communities could also help people feel less alone. For example, one participant observed that a key impact of an MMMV event was learning: ‘*We are not alone in this issue. It’s not only for our community… but it’s across the community that this issue is happening’* (Interview 9). They thought this normalization of mental health and wellbeing concerns may encourage help-seeking.

Similarly, some participants reported that MMMV had created an opportunity for between-generation communication within communities. These participants identified that there was a potential clash in understanding about mental health and wellbeing between parents who had immigrated to Australia and their children who had been born and/or raised here. Some of the participants had observed the positive impact of holding intergenerational community events, where young people, parents and elders listened to MMMV presenters together and were able to start conversations using those stories as an example. However, other participants noted the importance of also creating events and resources for specific groups of people, such as young people or women, to create a safe and engaging environment and to reach more diverse groups of people within their community.

*So hopefully the stigma is changing, even with the adults that were in the audience, they’re just hearing that if the kids have these needs, then we’re going to have to educate ourselves on how this system can support our children.* (Interview 1)

### The importance of safe and accessible language

Many interview participants, and some survey participants, were appreciative of being introduced to the concept of safe language when discussing mental health, wellbeing and suicide. By attending the training workshops, participants learned new ways to talk about mental health that were inclusive, respectful and did not cause harm to themselves or others. Some participants had found the knowledge useful beyond MMMV, using it in everyday conversation or when talking to family and friends about difficult issues.

*Keeping it authentic, but also making sure that the language I’m using is safe for other people. So I think that’s made it really easy for me in my daily life, to talk to my friends and my family and stuff about a difficult time without also triggering other people.* (Interview 4)

*It was important to learn safe language to use while addressing mental health illness in my community. The aim is to promote awareness and a safe space for people to normalise mental health discussions, empower people to address their own mental health and feel comfortable seeking support.* (Training Workshops Survey Participant 7)

Participants noted that language was an important consideration when working with multicultural communities. Language created barriers to accessing and using support services and was seen as a potential barrier to engaging with MMMV resources and events delivered in English. Some participants expressed enthusiasm for supporting future MMMV facilitators to train and present content in their own language. Participants noted that *‘…it would be easier for people to digest the information in their own language*’ (Interview 6) and that this approach would demonstrate genuine care for different cultures and communities. However, one participant noted that some people may feel safer talking about mental health and wellbeing in English rather than their own language. They recommended providing different options for people to choose from: *‘…the more options that you have, you will reach more people, and that is when you then truly embrace the multicultural landscape in Australia’* (Interview 5).

### Increased confidence and mental health literacy

Overall, participants agreed that the MMMV program gave them the confidence to deliver peer-led mental health education within their representative communities. Some participants felt more confident to reach out to their community to encourage action, e.g. supporting members of their community and connecting them with appropriate wellbeing resources ‘*it has always been my passion to bring health & wellbeing (Both Mental & Physical) awareness to the wider community & this workshop has given me more confidence & ideas of how to present this better to the larger community’* (Training Workshop Survey Participant 8), encouraging other people to connect with MIEACT, or asking for input into the development of new resources. However, when it came to implementing these skills in the MMMV program some participants would have liked to receive more support with planning, organizing and facilitating wellbeing events for their communities.

…*the problem is that for me personally, because of my lack of confidence, I needed more guidance. Because the tools that were given me was like OK, this is what we have, you can run it however you want. And that is quite scary. I wish that there were more materials developed, or someone hold my hand for the first time*. (Interview 5)

Participants also agreed that being part of MMMV had increased their mental health literacy. MMMV had created opportunities for starting conversations and taught participants about the language of mental health. Many participants discussed the way sharing stories of lived experience could start conversations or inspire other people to begin reflecting on their own mental health. Storytelling could break the ice on difficult topics, and set the tone of conversations, supporting people to feel safer and more comfortable talking about their own experiences.

*When somebody is being vulnerable, you have their sense of empathy and it also makes you think oh, I went through something like that. It just has a very deep sense, it just connects, … it’s normal, people do go through this … oh, if that person went through that, and I did, it’s OK. That this person was able to get help and support and of course that means I can too.* (Interview 3)

### Positive impacts on personal and community attitudes

Participants described the way the MMMV program had affected their personal attitudes, the ways they hoped the program would change community attitudes and the need for sufficient time for change to take effect. Some participants reported that attending the training workshops had broadened their own perspective on mental health and wellbeing. This was true even for participants with prior knowledge about these topics; the workshops still offered fresh viewpoints and information on less familiar topics.

*I already am exposed to health and wellbeing topics and the way these topics were handled and the extra resources provided definitely made me understand more of the different aspects of mental health and well being and has helped me seek more information.* (Training Workshops Survey Participant 15)

Being part of MMMV also had personal impacts on some participants; emotions and feelings were validated, and participants felt more valued and secure in their identity, and less alone in their experiences. Listening to other people’s lived experience stories was described as empowering and inspired some participants to reflect on their own wellbeing or see their past struggles in a different light.

…*my experience with My Mind, My Voice, talking to all these different people from different cultural backgrounds, it gave me the opportunity to actually take stock of my experience. And then it’s giving me the space to validate my own emotions, my own experiences, and then I can move on from the past. It actually opened that door for me, to move on, move beyond that shame and the pain that I experienced.* (Interview 5)

Participants thought that the MMMV program has the potential to improve attitudes towards mental health and wellbeing in their communities. Most participants agreed that participating in MMMV was influencing attitudes in their community regarding experiences of mental ill-health and would lead to increased acceptance and acknowledgement of wellbeing concerns in their community. Creating opportunities for sharing experiences and having open, honest discussions about mental health were seen as important ways to start breaking down stigmatized beliefs. The inclusion of lived experience in MMMV workshops and events humanized the experience of mental health issues, let individuals and families know they were not alone in their experiences and provided real-life examples of how to start seeking support. Participants felt the workshops and events developed through MMMV could help to reduce the shame around mental illness and provide hope for those experience mental health difficulties.

…after *the girls were presenting… I can hear the young people… agreeing to what the girls were sharing. I was just sitting there, and said wow, that’s really impacting on the young people because they are saying yes, yes, that’s happening to us also, what they were saying. And they were happy also, because the parents were there to hear that too.* (Interview 9)

To have the best impact, several participants highlighted that the MMMV program needed more time; changing attitudes and stigma were described as needing a long-term plan, not a short-term project. Continuation of MMMV over a longer period would allow the program to build the relationships and reputation needed to engage more communities and reach more people. Participants also saw value in the provision of longer-term support for currently engaged groups to continue the work they had started; developing events and resources, and upskilling more people to positively influence attitudes and lives in their communities.

*So I think a program such as My Mind, My Voice, needs to carry on for longer, to have the biggest impact, because you do need to take time to build those relationships and connections, and we as community members can do that slowly, the resource will not come through immediately, it’ll take time*. (Interview 3)

## DISCUSSION

This study aimed to explore the perceived impacts and value of the My Mind, My Voice program and the processes that led to those impacts. The MMMV evaluation reinforced Colucci *et al.*’s (2015) findings; being involved with a co-produced program that was culturally relevant, respectful and that enhanced people’s confidence and literacy was a positive experience for participants with a range of perceived impacts. Feeling respected, valued and validated when engaging with MIEACT staff and other program participants, helped participants feel empowered to develop and deliver mental health and wellbeing education in their community. They appreciated the opportunity to learn about mental health from multiple perspectives, share ideas with other program participants and develop skills to talk about mental health, wellbeing and suicide safely. In line with Blignault *et al.*’s (2009) suggestion that the co-production of multicultural strategies was essential, when implementing these skills, many participants felt MMMV provided a balance between freedom to develop tailored resources and events, and enough guidance to feel supported.

MMMV was thought to impact mental health literacy and attitudes towards wellbeing by starting conversations through storytelling. Consistent with the work conducted by Colucci et al. (2015) participants perceived storytelling as a culturally appropriate and particularly effective form of communication, which could create a sense of connection, empathy and trust, and encouraged engagement more effectively than presenting facts and figures alone. Sharing stories of lived experience in a safe manner could make people feel less alone and break the ice on difficult topics, supporting people to feel more comfortable talking about their own experiences. Storytellers could also role model effective help-seeking. This suggests that mental health promotion interventions incorporating positive contact with people with lived experience of mental ill-health may be particularly appropriate and effective in CALD community contexts (Griffiths *et al*., 2014).

The importance of language and the barrier of not being able to articulate properly in a second language (Wohler and Dantas, 2017) were highlighted. Participants advocated for changes to the program that could incorporate sessions and workshops in other languages. However, while translated resources and workshops conducted in other languages could be helpful, translating concepts like mental health and mental illness was not always straightforward. Having a number of options including using English was also encouraged to alleviate the pressure of finding equivalent non-stigmatized terms for mental health concepts that may not be available in all languages. These findings highlight the importance of choice and collaboration with the community when designing mental health promotion programs for CALD communities. For multi-language interventions and resources, it may be valuable to compare participant experiences and outcomes when engaging with content presented in different languages.

Participants perceived positive impacts, or the potential for longer-term impacts, in their communities as a result of engaging in MMMV. Consistent with prior research, breaking down the barriers or difficulties of different concepts and language used to describe mental health, (Colucci *et al.*, 2015; Wohler and Dantas, 2017), mental health literacy (Blignault *et al.*, 2009), the perception of the appropriateness of seeking help (Colucci *et al.*, 2015), perceived safety of community resources (Vera and Conner, 2007) and perceived discrimination (Telenta *et al.*, 2020) were seen as important but long-term goals. Expanding the diversity of communities involved has the added potential benefit of increasing connections and learnings between different CALD groups. During the evaluation, some participants observed that connecting different cultural groups through mental health education could reduce their sense of being alone in their challenges and experiences. Intergenerational connections could also promote shared understandings between parents and children who have been born and/or raised in different countries, alleviating some of the intergenerational pressures mentioned by Blignault *et al.* (2022). Demographic information was not collected in this study due to concerns around participant confidentiality, so we cannot comment on the representativeness of the evaluation or program participants. Participants acknowledged that having an effect on attitudes around mental health would take time and may not necessarily be straightforward, continuing the funding of MMMV and similar programs would be one small step in achieving this long-term goal. Sustainable, long-term support of mental health promotion in CALD communities is likely to be a key element in achieving meaningful change.

As MMMV events and resources continue to be developed and implemented by partner organizations, it will be important to gather feedback on the impact and acceptability of the resources and experiences of those using the resources. Most participants in this evaluation had attended a DoNOHarm and/or Story Crafting training workshop, organized or developed a community event or resource or presented at an event. One of the limitations of the evaluation is the limited input from the viewpoint of people who had attended a MMMV community event, and no information was available for those who had accessed online resources. These groups should be key targets in future evaluation and quality improvement work. Given the challenges of recruiting event attendees in this evaluation, low impact, in-the-moment approaches to collecting feedback may be the most successful. It is also important to consider our participants’ reflections that changes in attitudes around mental health are a long-term goal. Gathering the evidence required to understand these changes will likely require long-term follow-up in evaluations. Community collaboration and co-evaluation may improve the feasibility of collecting data at multiple time points.

## CONCLUSION

The evaluation of the MMMV program provides evidence supporting the value and acceptability of community-led co-production of mental health and wellbeing education resources in Australian CALD communities. Participant feedback for MMMV was overwhelmingly positive and supported the success of the program in providing a safe and culturally inclusive space to learn, create and empower communities to find ways to promote mental health and wellbeing. Genuine partnership with CALD organizations, balancing capacity building and community needs, should be a key focus for the future development and implementation of health promotion initiatives in CALD communities. Successful co-design and implementation processes have the potential to open the channels of communication both within and between communities. Open, honest conversations about mental health and wellbeing are an important way to break down stigma and start conversations about mental health and wellbeing in CALD communities.

While MMMV was a positive experience for organization and community members who chose to engage in developing mental health promotion content, evidence of the acceptability, value and impact of this content in the broader CALD community is needed. Brief, collaboratively designed evaluation methods may be the most feasible approach to gathering this evidence in future mental health promotion initiatives. In this study, participants’ constructive feedback typically centred on ways to extend or continue the work of MMMV, indicating continuing demand for the program in the ACT. Changing attitudes and stigmatized beliefs regarding mental illness are long-term goals, which will likely require the sustained funding and implementation of effective mental health promotion initiatives. MMMV provides an example of a culturally safe and respectful approach to co-designing and implementing such initiatives in an Australian context.

## Data Availability

The data underlying this article cannot be shared publicly due to ethical requirements.
